# The Mitochondrial Genomes of *Aquila fasciata* and *Buteo lagopus* (Aves, Accipitriformes): Sequence, Structure and Phylogenetic Analyses

**DOI:** 10.1371/journal.pone.0136297

**Published:** 2015-08-21

**Authors:** Lan Jiang, Juan Chen, Ping Wang, Qiongqiong Ren, Jian Yuan, Chaoju Qian, Xinghong Hua, Zhichun Guo, Lei Zhang, Jianke Yang, Ying Wang, Qin Zhang, Hengwu Ding, De Bi, Zongmeng Zhang, Qingqing Wang, Dongsheng Chen, Xianzhao Kan

**Affiliations:** 1 The Institute of Bioinformatics, College of Life Sciences, Anhui Normal University, Wuhu, Anhui, China; 2 The Provincial Key Laboratory of the Conservation and Exploitation Research of Biological Resources in Anhui, Wuhu, Anhui, China; 3 The College of Life Sciences, Peking University, Beijing, China; 4 Key Laboratory of Stress Physiology and Ecology in Cold and Arid Regions, Department of Ecology and Agriculture Research, Cold and Arid Regions Environmental and Engineering Research Institute, Chinese Academy of Sciences, Lanzhou, Gansu, China; 5 The Ningguo Museum of Natural History, Ningguo, Anhui, China; 6 The Department of Medical Biology, Wannan medical college, Wuhu, Anhui, China; Sichuan University, CHINA

## Abstract

The family Accipitridae is one of the largest groups of non-passerine birds, including 68 genera and 243 species globally distributed. In the present study, we determined the complete mitochondrial sequences of two species of accipitrid, namely *Aquila fasciata* and *Buteo lagopus*, and conducted a comparative mitogenome analysis across the family. The mitogenome length of *A*. *fasciata* and *B*. *lagopus* are 18,513 and 18,559 bp with an A + T content of 54.2% and 55.0%, respectively. For both the two accipitrid birds mtDNAs, obvious positive AT-skew and negative GC-skew biases were detected for all 12 PCGs encoded by the H strand, whereas the reverse was found in MT-ND6 encoded by the L strand. One extra nucleotide‘C’is present at the position 174 of MT-ND3 gene of *A*. *fasciata*, which is not observed at that of *B*. *lagopus*. Six conserved sequence boxes in the Domain II, named boxes F, E, D, C, CSBa, and CSBb, respectively, were recognized in the CRs of *A*. *fasciata* and *B*. *lagopus*. Rates and patterns of mitochondrial gene evolution within Accipitridae were also estimated. The highest dN/dS was detected for the MT-ATP8 gene (0.32493) among Accipitridae, while the lowest for the MT-CO1 gene (0.01415). Mitophylogenetic analysis supported the robust monophyly of Accipitriformes, and Cathartidae was basal to the balance of the order. Moreover, we performed phylogenetic analyses using two other data sets (two mitochondrial loci, and combined nuclear and mitochondrial loci). Our results indicate that the subfamily Aquilinae and all currently polytypic genera of this subfamily are monophyletic. These two novel mtDNA data will be useful in refining the phylogenetic relationships and evolutionary processes of Accipitriformes.

## Introduction

Bonelli’s eagle (*Aquila fasciata*) and Rough-legged buzzard (*Buteo lagopus*) are birds of prey of the hawk and eagle family (Accipitridae). *A*. *fasciata*, which previously was a member of the genus *Hieraaetus*, has a fragmented distribution from Mediterranean basin to India, south of China, and Indochina [1]. *B*. *lagopus*, also called Rough-legged hawk, is a widespread bird of prey. In China, these two species are protected species, and listed in the first category of National Key Protected Wild Animals. The family Accipitridae (hawks, eagles, and kites), with other four families (Cathatidae, Pandionidae, Sagittariidae, and Falconidae), was traditionally classified within the order Falconiformes[2–4]. The monophyly of Falconiformes was supported by some morphological classifications[4–6]. However, recent comprehensive genetic analyses disprove any close phylogenetic relationship between the Falconidae and other families included in the order Falconiformes [7–10]. Based on the proposals of Hackett et al.[8] and the South American Classification Committee(SACC), the Falconiformes is now restricted to include only the family Falconidae, and the remaining four families are put in the separate order Accipitriformes[1,11,12]. The family Accipitridae is one of the largest groups of non-passerine birds, including 68 genera and 243 species globally distributed [1]. Within the 68 genera of Accipitridae, 36 are monotypic, and 32 are polytypic. This family has been variously divided into three to fourteen subfamilies[13–17]. Peters(1931)[16] recognized eight subfamilies within Accipitridae. Lerner and Mindell (2005) [17] accepted that the Accipitridae consisted of fourteen subfamilies (Accipitrinae, Aegypiinae, Aquilinae, Buteoninae, Circaetinae, Circinae, Elaninae, Gypaetinae, Haliaeetinae, Harpiinae, Melieraxinae, Milvinae, Perninae, and Polyboroidinae). Griffiths et al. (2007) [14] found that the family included eight major lineages (Elanini, Gypaetina, Pernina, Circaetina, Gypina, Harpiita, Aquilita, and Accipitrita), which they named using tribe, subtribe, and infratribe ranks.

The aquiline eagles (Aquilinae), one of the largest accipitrid groups, traditionally include 35–36 species in six monotypic genera (*Ictinaetus*, *Lophaetus*, *Oroaetus*, *Polemaetus*, *Spizastur*, and *Stephanoaetus*) and three polytypic genera (*Aquila*, *Hieraaetus*, and *Spizaetus*) [17]. There is no doubt about the monophyly of aquiline eagles [13,18]. However, all three polytypic genera (*Aquila*, *Hieraaetus*, and *Spizaetus*) turned out to be paraphyletic[14,17–21]. The three spotted eagles (*A*. *clanga*, *A*. *hastata*, and *A*. *pomarina*) were treated as a new genus *Clanga* [22,23], which formed a clade sister to *Ictinaetus* and to *Lophaetus*. Because *Spizastur* merged into *Spizaetus* [18,24], the former should be moved to the latter [1,12]. Based on the phylogenetic analysis of MT-CYB (mitochondrially encoded cytochrome b) and control region sequences, Haring et al. [24] proposed the following four suggestions: (1) the monotypic genus *Oroaetus* should be moved to *Spizaetus*, (2) the Old World species of *Spizaetus* were separated into *Nisaetus*, (3) Cassin’s hawk-eagle was moved from *Spizaetus* to *Aquila*, and (4) Rufous-bellied hawk-eagle was separated from *Hieraaetus* into the monotypic genus *Lophotriorchis*. So, in the present study, we recognize aquiline eagles as 39 species in five monotypic genera (*Ictinaetus*, *Lophaetus*, *Lophotriorchis*, *Polemaetus*, and *Stephanoaetus*) and five polytypic genera (*Aquila*, *Clanga*, *Hieraaetus*, *Nisaetus*, and *Spizaetus*), respectively. The extensive molecular data, with full complete taxonomic representation, are urgently needed to reassess the monophyly of currently polytypic genera of Aquiline eagles. Typical metazoan mitochondrial genomes are double-stranded, circular molecules, 16–20 kb in length, and typically include 13 protein-coding genes, 22 tRNA genes, two rRNA genes, as well as one control region (CR). Compared with the nuclear genome, the mitochondrial genome has several intrinsic characteristics, such as small genome, maternal inheritance mode and fast substitution rate. Mitochondrial genomes have been proposed to show potential in resolving ancient patterns of evolutionary history [25]. Recently, a number of studies performed using mitochondrial genome sequences to elucidate avian phylogeny[25–35]. For example, Here, we present the complete mitochondrial genomes of Bonelli’s eagle and Rough-legged hawk. Based on new data generated from *A*. *fasciata*, *B*. *lagopus* and existing sequence data from GenBank, we tried to address: (1) features of the mitogenomes of *A*. *fasciata* and *B*. *lagopus*, (2) rates and patterns of mitochondrial gene evolution within Accipitridae, and (3) the monophyletic status of currently recognized polytypic genera of Aquilinae.

## Materials and Methods

### Sample collection and DNA extraction

The frozen muscle tissue samples of *A*. *fasciata* (code Kan-K0318) and *B*. *lagopus* (code Kan-K0381) were provided by the Ningguo Museum of Natural History (NMNH), Anhui Province, China. NMNH is authorized to collect specimens. Tissues were stored at -20°C at the College of Life Sciences, Anhui Normal University, China. Total genomic DNA of these specimens were extracted from the muscle tissue following the method of Sambrook and Russell (2001) [36].

### PCR amplification and sequencing

The PCR primers and several internal primers (S1 Table) used in PCR amplification or sequencing were designed based on available mitochondrial sequences of Accipitriformes. Each primer set amplified a mtDNA fragment, including an overlap region of at least 100 bp with its adjacent amplified fragments at both the terminals. Long PCR and nested-PCR were performed as described by Kan et al. [28]. The amplified fragments were purified using TIANgel Midi Purification Kit (Tiangen Biotech Co., Ltd, Beijing, China). The purified PCR products were sequenced directly on ABI-PRISM 3730 sequencer using BigDye Terminator v3.1 Cycle Sequencing Kit (Applied Biosystems) with their corresponding primers.

### Genome assembly and annotation

DNA sequences were analyzed using software BioEdit [37] and Ugene [38]. Contig assembly was performed with the program Sequencher 4.14 (Gene Codes Corporation, Ann Harbor, USA). The boundaries of protein-coding genes and rRNA genes were initially identified via the MITOS [39] and DOGMA [40] webservers, and refined by alignment with mitochondrial genomes of other species of Accipitriformes. Transfer RNA genes were identified using tRNAscan-SE v.1.21 [41] and ARWEN v.1.2 [42]. The whole-mtgenome comparison maps were visualized using the software CGView Comparison Tool (CCT) [43]. All gene names included mitochondrial and nuclear gene are in accordance with the HUGO Gene Nomenclature Committee’s database (HGNC) [44].

### Sequence alignment and Rate heterogeneity

Sequence alignment was carried out using MAFFT 7.2 [45] with the default settings. The nucleotide bias, skew can be calculated as (G − C) / (G + C) or (A − T) / (A + T). The rates (number of variable sites, ratio of nonsynonymous-to-synonymous substitutions rates (dN/dS)) and patterns (Transition-to-transversion (ts/tv) ratio) of evolution for each gene were calculated in the present study. Number of variable sites was conducted using DnaSP ver. 5.10 [46]. dN/dS was performed with Datamonkey[47]. ts/tv was estimated by MEGA ver. 6.06 [48].

### Phylogenetic analysis

To investigate the evolutionary relationships among *A*. *fasciata*, *B*. *lagopus* and their related species, three data sets were performed with the maximum likelihood (ML) and the Bayesian inference (BI) methods: (1) for mitogenomic phylogeny of Accipitriformes data set, 13 PCGs of 16 Accipitriformes species were used (Table 1), with two species from Strigiformes (*Phodilus badius*, NC_023787; *Strix leptogrammica*, NC_021970) as the outgroups,(2) for phylogeny of Accipitridae data set, two mitochondrial genes (MT-CYB and MT-ND2 (mitochondrially encoded NADH dehydrogenase 2)) of 148 species from Accipitridae were used (S2 Table), and (3) for phylogeny of Aquilinae data set, available multiple sequences of four mtDNA loci (MT-CYB, MT-ND2, MT-CO1 (mitochondrially encoded cytochrome c oxidase I) and CR) and five nuclear loci (RAG1 (recombination activating gene 1) coding region, LDH (lactate dehydrogenase) intron 3, MYC (v-myc avian myelocytomatosis viral oncogene homolog), AK1 (adenylate kinase 1) exon 6, FIB7 (beta-fibrinogen gene, intron 7)) of all 39 species in this subfamily from GenBank were used (S3 Table), with *Morphnus guianensis* and *Harpia harpyja* as the outgroups. Codon positions included in the analysis were the 1st, 2nd and 3rd. Sequence alignment was carried out using MAFFT 7.2 [45] with the default settings. Sequence format convertion was performed with DAMBE 5.5 [49]. To check for saturation in nucleotide codons, substitution saturation analysis [50] was performed for subsets with the first, second and third codon positions using DAMBE 5.5. According to the results, none of the substitutions from three codon positions of all protein-coding genes in our two data sets were saturated. The best-fit models were selected using Bayesian Information Criterion (BIC) as implemented in ModelGenerator version 0.85[51]. For 13 PCGs mitogenome nucleotides data set, we defined the independent mitochondrial partitions as each of the 13 loci. For combined mitochondrial and nuclear data set, we defined independent partitions as each of the 9 loci.

**Table 1:** Species of mitogenomes examined in this study as classified according to Clements *et al*. (2014).

Species	Family	Accession no.	Reference
*Accipiter gentilis* (Northern Goshawk)	Accipitridae	NC_011818	[73]
*Accipiter nisus* (Eurasian Sparrowhawk)	Accipitridae	NC_025580	[65]
*Accipiter soloensis* (Chinese Sparrowhawk)	Accipitridae	KJ680303	Unpublished data^a^
*Accipiter virgatus* (Besra)	Accipitridae	NC_026082	[34]
*Aegypius monachus* (Cinereous Vulture)	Accipitridae	NC_022957	Unpublished data^b^
*Aquila chrysaetos* (Golden Eagle)	Accipitridae	NC_024087	[66]
*Aquila fasciata* (Bonelli’s Eagle)	Accipitridae	KP329567	This study
*Buteo buteo* (Common Buzzard)	Accipitridae	NC_003128	[27]
*Buteo buteo burmanicus* (Common Buzzard (Himalayan))	Accipitridae	KM364882	[67]
*Buteo lagopus* (Rough-legged Hawk)	Accipitridae	KP337337	This study
*Nisaetus alboniger* (Blyth’s Hawk-Eagle)	Accipitridae	NC_007599	[68]
*Nisaetus nipalensis* (Mountain Hawk-Eagle)	Accipitridae	NC_007598	[68]
*Spilornis cheela* (Crested Serpent-Eagle)	Accipitridae	NC_015887	Unpublished data^c^
*Cathartes aura* (Turkey Vulture)	Cathartidae	NC_007628	[33]
*Pandion haliaetus* (Osprey)	Pandionidae	NC_008550	[26]
*Sagittarius serpentarius* (Secretary-bird)	Sagittariidae	NC_023788	[10]

Note: Unpublished data:

^a^ Lee,Y.J., Ryu,S.H. and Hwang,U.W;

^b^ Li B and Zhou L;

^c^ Qin X, Shi J, Guan Q, et al.

The ML analyses were conducted in RAxML v.8.0.26 [52], as implemented in the graphical user interface RaxML GUI v.1.3.1 [53]. We performed analyses with ML + slow bootstrap for ten runs and 1000 replicates under GTR + CAT [54] model. Due to lower computational and memory costs, CAT, which is a rate heterogeneity and fast approximation of the gamma model, appears to yield better log likelihood scores even when calculated under a real gamma model [54,55]. Under the GTR + CAT model, the default number of categories (c = 25) and random starting trees were employed.

The BI analyses were performed with MrBayes 3.2.2 [56], applying independent models of evolution for each partition. For 13 PCGs nucleotides data set, GTR + G was chosen for MT-ND4 (mitochondrially encoded NADH dehydrogenase 4), HKY+I+G was chosen for MT-ND1 (mitochondrially encoded NADH dehydrogenase 1), HKY+G was chosen for MT-ATP8 (mitochondrially encoded ATP synthase 8), MT-ND3 (mitochondrially encoded NADH dehydrogenase 3) and MT-ND4L (mitochondrially encoded NADH dehydrogenase 4L), while GTR + I + G for the remaining 10 loci. For combined mitochondrial and nuclear data set, GTR + I + G was chosen for MT-CYB, MT-ND2, and MT-CO1, while GTR + G for the remaining six loci were selected as the best substitution model for the 1st, 2nd, and 3rd codon positions, respectively. Four Markov chains were run for ten million generations (sampling every 100 generations) allowing adequate time for convergence. After discarding 25% of the initial trees as burn-in, the remaining trees were used to estimate 50% majority rule consensus tree and Bayesian posterior probabilities (BPP). All MCMC runs were repeated twice to confirm consistent approximation of the posterior parameter distributions. PSRF (potential scale reduction factor) is close to 1 as runs converge, and the ESS (effective sample sizes) for all parameters are above 200.

## Results and Discussion

### Features of the mitogenomes of *A*. *fasciata* and *B*. *lagopus*

**Fig 1:**
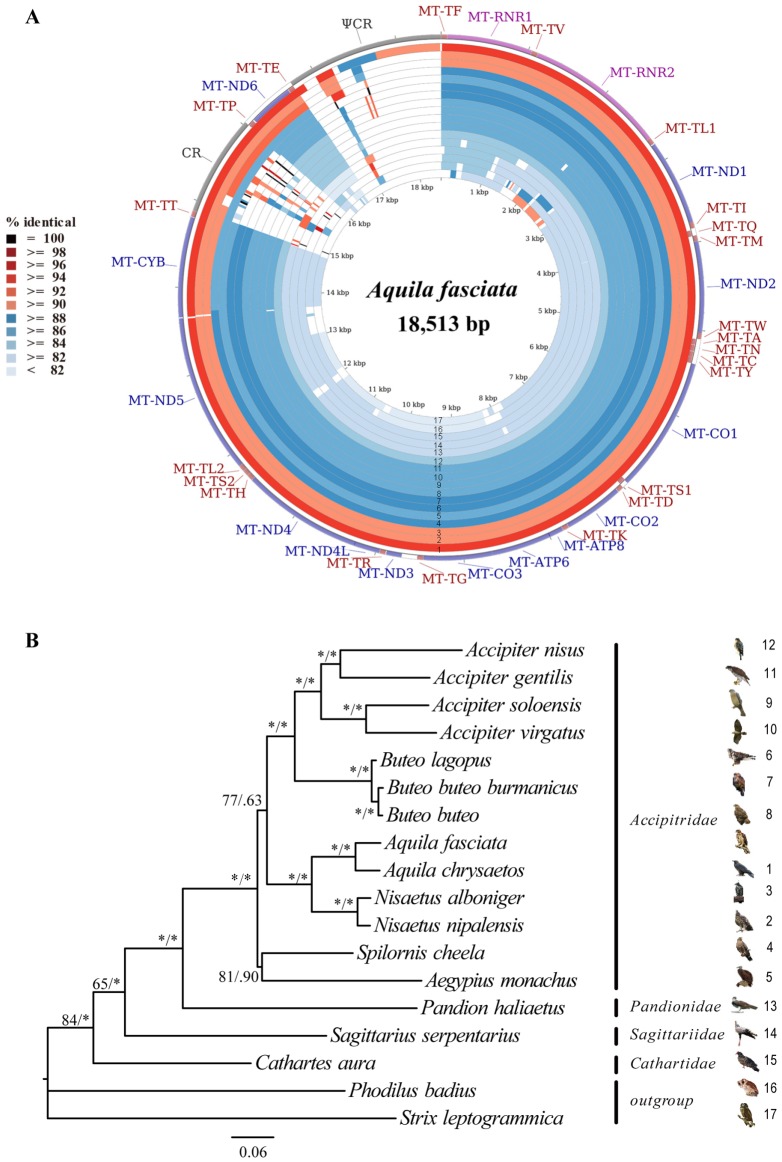
(A) Graphical map of the BLAST results showing nucleotide identity between *A*. *fasciata* mitogenome and 15 related species listed in Table 1, as generated by the CGView comparison tool (CCT). CCT arranges BLAST result in an order where sequence that is most similar to the reference (*A*. *fasciata*) is placed closer to the outer edge of the map. The rings labelled 1 to17 indicate BLAST results of *A*. *fasciata* mitogenome against *A*. *chrysaetos*, *N*. *nipalensis*, *N*. *alboniger*, *S*. *cheela*, *A*. *monachus*, *B*. *lagopus*, *B*. *buteo*, *B*. *buteo burmanicus*, *A*. *soloensis*, *A*. *virgatus*, *A*. *gentilis*, *A*. *nisus*, *P*. *haliaetus*, *S*. *serpentarius*, *C*. *aura*, *P*. *badius*, and *S*. *leptogrammica*, respectively. (B) Nucleotide-based phylogenetic tree of 16 Accipitriformes species, with two Strigiformes birds as outgroups. This analysis is based on 13PCGs. Both ML and Bayesian analyses produced identical tree topologies. The ML bootstrap and Bayesian posterior probability values for each node are indicated.

**Fig 2:**
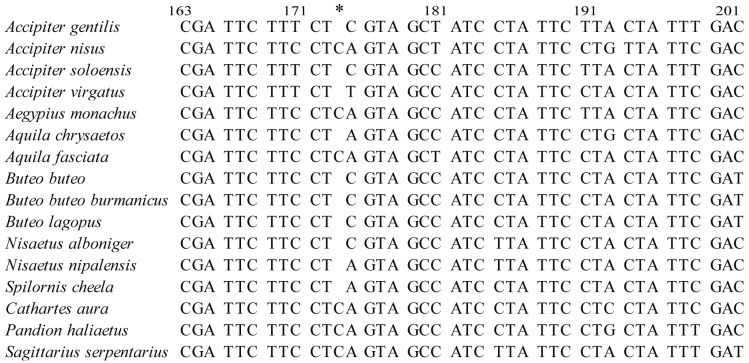
Alignment of the mitochondrial MT-ND3 gene region spanning the extra base site (designated by *) for 16 Accipitriformes species. MT-ND3 for *A*. *nisus*, *A*. *monachus*, *A*. *fasciata*, *C*. *aura*, *P*. *haliaetus*, *S*. *serpentarius* with and for the other 10 species without the extra base.

The complete mitochondrial genomes (mt-genomes) of *A*. *fasciata* and *B*. *lagopus* were determined to be 18,513 and 18,559 bp in length, respectively. These are close to the other Accipitriformes mt-genomes sizes reported (S4 Table). The two sequences were deposited in GenBank (*A*. *fasciata*: KP329567 and *B*. *lagopus*: KP337337). The number and order of 37 mitochondrial genes and two control regions (CR and ΨCR (pseuo control region)) of both *A*. *fasciata* and *B*. *lagopus* are identical (Fig 1A, and S5 Table). The nucleotide compositions of the complete mtDNA sequences (Heavy-strand) of *A*. *fasciata* and *B*. *lagopus* are slightly biased toward A and T (S4 Table), which is similar to that from other avian species[28,29,35]. Comparative analyses of the nucleotide sequences of each mt gene (excluded tRNA genes) and non-coding regions, together with the amino acid sequences, are given in Table 2. The sequence lengths of 13 PCGs were the same for *A*. *fasciata* and *B*. *lagopus*, except for MT-ND3 gene (Table 2). One extra nucleotide ‘C’ is present at the position 174 of MT-ND3 gene of *A*. *fasciata*, which is not observed at that of *B*. *lagopus*. In mitochondrial MT-ND3 for 16 species of Accipitriformes (Fig 2), 6 have the extra base, whereas 10 lack it. Additional putative frameshift sites have been proposed in the mitochondrial of many birds, turtles and ants[28,29,35,57–59]. Mindell el al. [58] suggested that the site was subject to a +1 programmed frameshit during translation, allowing correct translational of functional protein. We also found that the extra base insertion appear to be dynamically gained and lost from the MT-ND3 of closely related taxa, such as the MT-ND3 of *A*. *fasciata* and *A*. *chrysaetos*. So, we infered that there was no evolutionary significance for the extra insertion site.

**Table 2:** Differences in mitochondrial nucleotides and predicted amino acids sequences between *Aquila fasciata* (AF) and *Buteo lagopus* (BL).

Gene/region	Nucleotide sequence length (bp)		Nucleotide difference (%)	Number of aa		aa difference (%)
	AF	BL	AF/BL	AF	BL	AF/BL
MT-ND1	978	978	13.50	325	325	4.92
MT-ND2	1041	1041	13.45	346	346	10.69
MT-ND3	352	351	10.83	116	116	3.45
MT-ND4	1380	1380	10.94	459	459	3.49
MT-ND4L	297	297	12.79	98	98	7.14
MT-ND5	1818	1818	13.59	605	605	10.25
MT-ND6	519	519	14.64	173	173	6.98
MT-CO1	1551	1551	11.35	516	516	11.63
MT-CO2	684	684	10.67	227	227	3.52
MT-CO3	786	786	9.54	261	261	4.98
MT-ATP6	684	684	14.62	227	227	5.73
MT- ATP8	168	168	22.02	55	55	34.55
MT- CYB	1143	1143	12.16	380	380	4.47
MT- RNR1	971	973	9.93			
MT- RNR2	1594	1594	12.08			
CR	1159	1654	51.44			
ΨCR	1799	1360	50.19			

**Fig 3:**
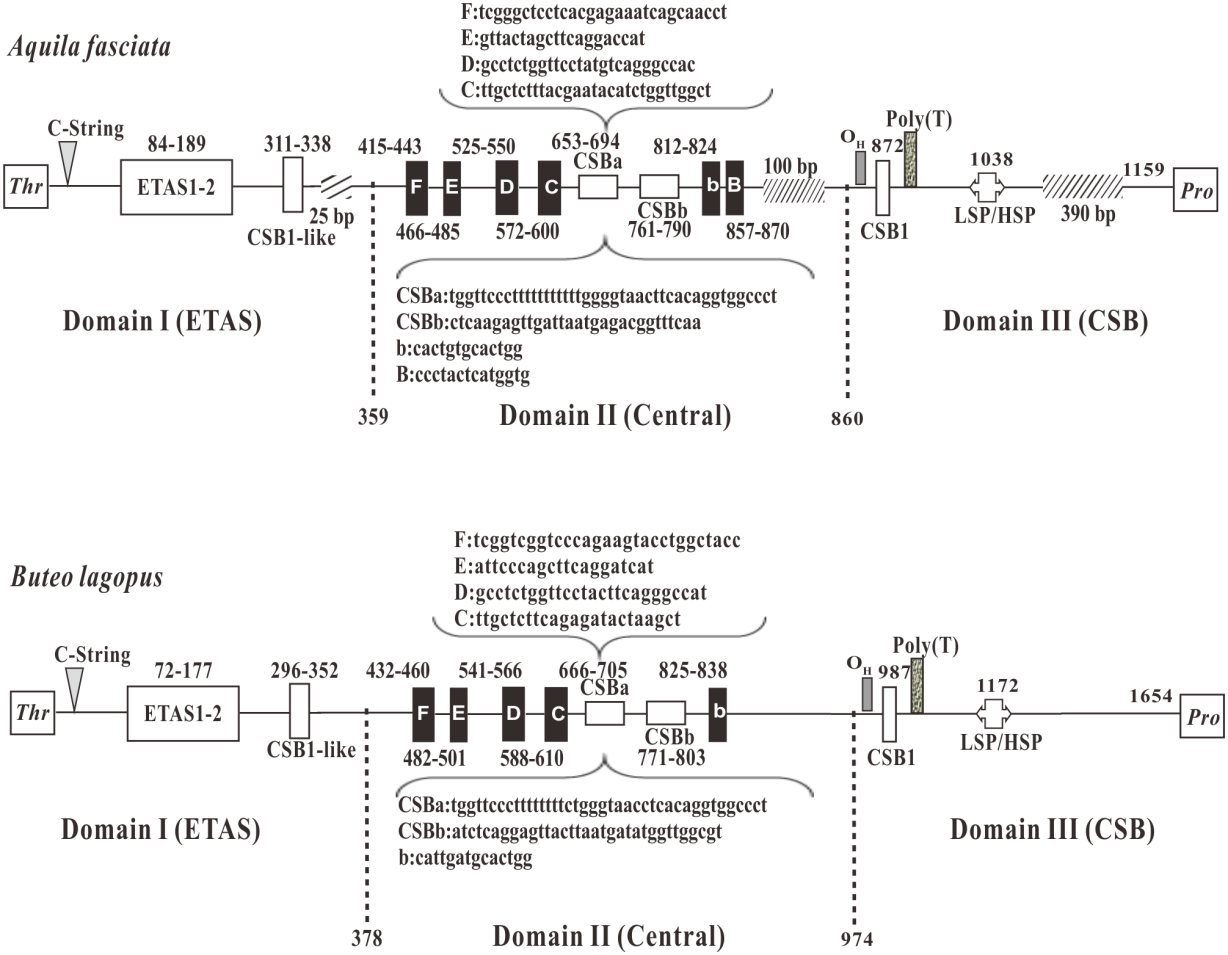
Base composition of *A*. *fasciata* and *B*. *lagopus* mitochondrial genomes. AT skew and GC skew are calculated for each protein-coding gene and other gene regions.

In addition, for both *A*. *fasciata* and *B*. *lagopus* mtDNAs, obvious positive AT-skew and negative GC-skew biases were detected for all 12 PCGs encoded by the H strand, whereas the reverses were found in MT-ND6 (mitochondrially encoded NADH dehydrogenase 6) encoded by the L strand (Fig 3). The nucleotide variation across the entire genome sequence between the two accipitrid species was 19.07%. The values of nucleotide sequence difference in 13 PCGs and two RNA genes between *A*. *fasciata* and *B*. *lagopus* ranged from 9.54% (MT-CO3 (mitochondrially encoded cytochrome c oxidase III)) to 22.02% (MT-ATP8) (Table 2). The amino acid sequence differences ranged from 3.45 to 34.55%, with MT-ND3 being the most conserved PCG and MT-ATP8 the least conserved. Like other Accipitriformes species (expect for *Cathartes aura* and *Sagittarius serpentarius*) [34,60,61] (S4 Table), two putative control regions (CR and ΨCR) of *A*. *fasciata* and *B*. *lagopus* mitogenomes were found between MT-TT (mitochondrially encoded tRNA threonine) and MT-TF (mitochondrially encoded tRNA phenylalanine), and were separated by MT-TP (mitochondrially encoded tRNA proline), MT-ND6 and MT-TE (mitochondrially encoded tRNA glutamic acid) (Fig 1A). The length of CR of Accipitriformes species varies between 1,117 bp (*S*. *serpentarius*) and 2,329 bp (*Accipiter nisus*), with AT content ranging from 55.9% (*A*. *monachus* and *Spilornis cheela*) to 65.0% (*A*. *nisus*) (S4 Table).

**Fig 4:**
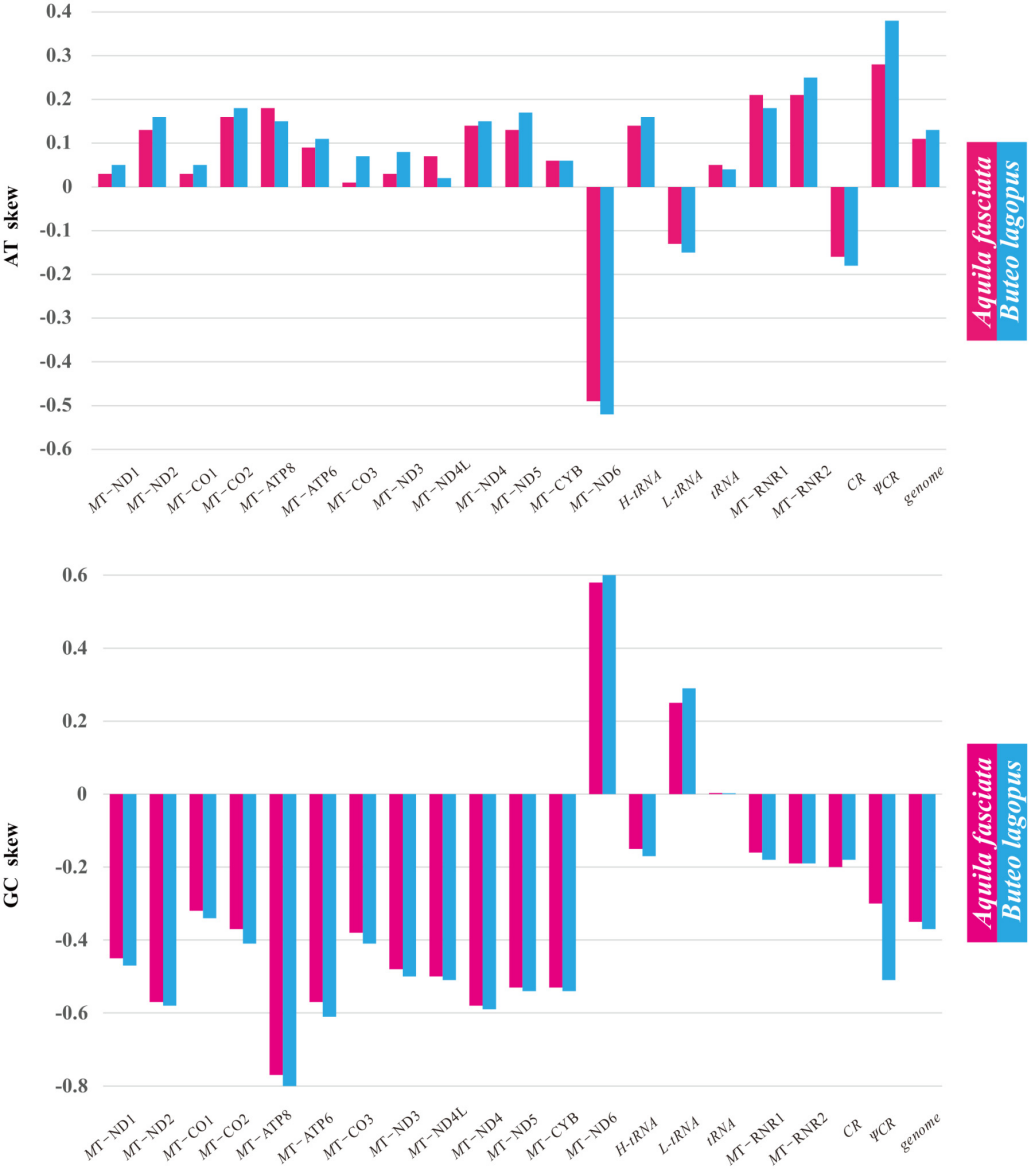
The structures of control region (CR) in mtDNA of *A*. *fasciata* and *B*. *lagopus*. Positions of the conserved boxes and the division into the three domains Domain I (ETAS), Domain II (Central), Domain III (CSB) are shown. ETAS = extended termination-associated sequences; F through B boxes = conserved sequence boxes in the central domain, CSBa is highly conserved stretches that vary in length, while CSBb is more variable; CSB = conserved sequence block; CSB-like = a sequence similar to the CSB; LSP = light-strand transcription promoter; HSP = heavy-strand transcription promoter; twill box means the comparison of two CRs, the lack of base number.

Based on the distribution of the conserved motifs in other avian CRs [29,35,62,63], the CRs of the two accipitrid species could be divided into three domains: ETAS (extended termination-associated sequence) Domain I, Central Conserved Domain II and CSB (conserved sequence block) Domain III. Six conserved sequence boxes in the Domain II [60], named boxes F, E, D, C, CSBa, and CSBb, respectively, were recognized in the CRs of *A*. *fasciata* and *B*. *lagopus*. Nevertheless, conserved sequence box B was only found in the CR of *A*. *fasciata* (Fig 4). In the CR of *B*. *lagopus*, we observed three minisatellites, one was 23 nucleotides (5′-TTTATCATCATATTTTATTATTA) with six tandem repeats, and two were 11 nucleotides (5′-AAATTTTTACA, and 5′- AATTTATCATG) with 3 and 17 tandem repeats, correspondingly. Moreover, we only found one minisatellite, which was 22 nucleotides (5′-TTTTTTCACAATTTTTTCACAT) with two tandem repeats, in the CR of *A*. *fasciata*. The size, repeats, and sequence composition of minisatellite in Accipitriformes CRs might be served as useful genus- and species-specific molecular markers.

### Rates and patterns of mitochondrial gene evolution within Accipitridae

As can be seen in the CCT BLAST map, CR and ΨCR of these accipitrids are highly divergent (Fig 1A). Comparison of the evolutionary rate of different genes provides a better understanding of the patterns of molecular evolution of the mtDNA. Due to the length and sequence heterogeneity, CR and ΨCR sequences were not included in these analyses. Among the eleven mtDNA sequences (excluding CR and ΨCR), 5213 nucleotides sites (33.28%) are variable (Table 3). In the protein coding region, the most variable region of the genomes by percent variable sites is MT-ND3, followed by, MT-ATP8, MT-ND4 and MT-ND6. In contrast, the lowest one is tRNA genes (20.19%), followed by MT-RNR1 (mitochondrially encoded 12S RNA) and MT-RNR2 (mitochondrially encoded 16S RNA).

**Table 3:** Rates and patterns of evolution among mitochondrial genes and species of Accipitridae.

Gene	Length (bp)	Var. sites (%)	dN/dS	ts/tv
MT-RNR1	1006	253(25.15)	-	4.132
MT-RNR2	1663	416(25.02)	-	3.728
MT-ND1	978	344(35.17)	0.04345	3.854
MT-ND2	1047	431(41.17)	0.08984	3.897
MT-CO1	1551	456(29.40)	0.01546	4.611
MT-CO2	684	211(30.84)	0.03350	4.301
MT-ATP8	168	85(50.60)	0.29896	5.097
MT-ATP6	684	250(36.55)	0.06321	3.547
MT-CO3	786	244(31.04)	0.03582	3.378
MT-ND3	351	126(35.90)	0.07592	2.857
MT-ND4L	297	116(39.06)	0.06823	4.848
MT-ND4	1386	641(46.23)	0.08683	2.426
MT-ND5	1818	668(36.74)	0.07362	2.985
MT-CYB	1152	444(38.54)	0.05494	2.242
MT-ND6	519	210(40.46)	0.10653	4.093
tRNAs	1575	318(20.19)	-	6.640
Overall	15665	5213 (33.28)	-	3.404

Note: Var. sites = variable sites, dN = nonsynonymous rate, dS = synonymous rates, ts/tv = transititon-to-transversion ratio.

The ts/tv in the concatenated nucleotide data varies from MT-CYB (2.242) to *tRNA*s (6.640) (Table 3). As we know, transversions are typically rare in the slow-evolving regions, such as tRNA genes. However, the patterns (ts/tv) among protein-coding genes are variable and do not reflect differences in either synonymous or nonsynonymous rates among the genes [31,64]. For example, high ts/tv ratios were found for MT-CO1, MT-CO2 (mitochondrially encoded cytochrome c oxidase II), and MT-ND4L genes, which have intermediate rates in both these substitution categories. Furthermore, the highest dN/dS was detected for the MT-ATP8 gene (0.29896) among Accipitridae, while the lowest for the MT-CO1 gene (0.01546).

### Phylogenetic analysis

To infer mitophylogenetic relationships among accipitrids, evolutionary analysis was carried out using 13PCGs of 16 species, with two species of Strigiformes as outgroups. The ML and BI trees showed identical topologies (Fig 1B) [65–68]. The monophyly of Accipitridae was strongly supported (Fig 1B). New World vultures (Cathartidae) have some characters in common with storks, and a close phylogenetic relationship with storks was suggested [69–72]. However, recent molecular studies demonstrated that the New World vultures clearly have their affinity with other raptors and not with storks[7,8,21]. Our phylogeny indicated that *C*. *aura* (Cathartidae) was basal to the remaining of Accipitriformes. Based on relatively short sequences, Wink and Sauer-Gurth [21] placed Secretarybird (*S*. *serpentarius*) with storks. In our analysis, the Secretarybird is deepest on the branch with Osprey (*Pandion haliaetus*) and Accipitridae(Fig 1B). This result is in congruence with more and more recent studies, such as Lerner and Mindell[17], Mahmood et al.[10] and Hackett et al.[8]. Furthermore, *P*. *haliaetus* (Pandionidae) is the sister one to Accipitridae. The relationships among the four families of Accipitriformes is consistent with Burleigh et al.’ s results [7]. There were three clades in Accipitridae. One was (*Aegypius* + *Spilornis*), one was (*Aquila* + *Nisaetus*), and the other was (*Accipiter* + *Buteo*). *Aegypius* is a member of Aegypiinae, while *Spilornis* is that of Circaetinae.

**Fig 5:**
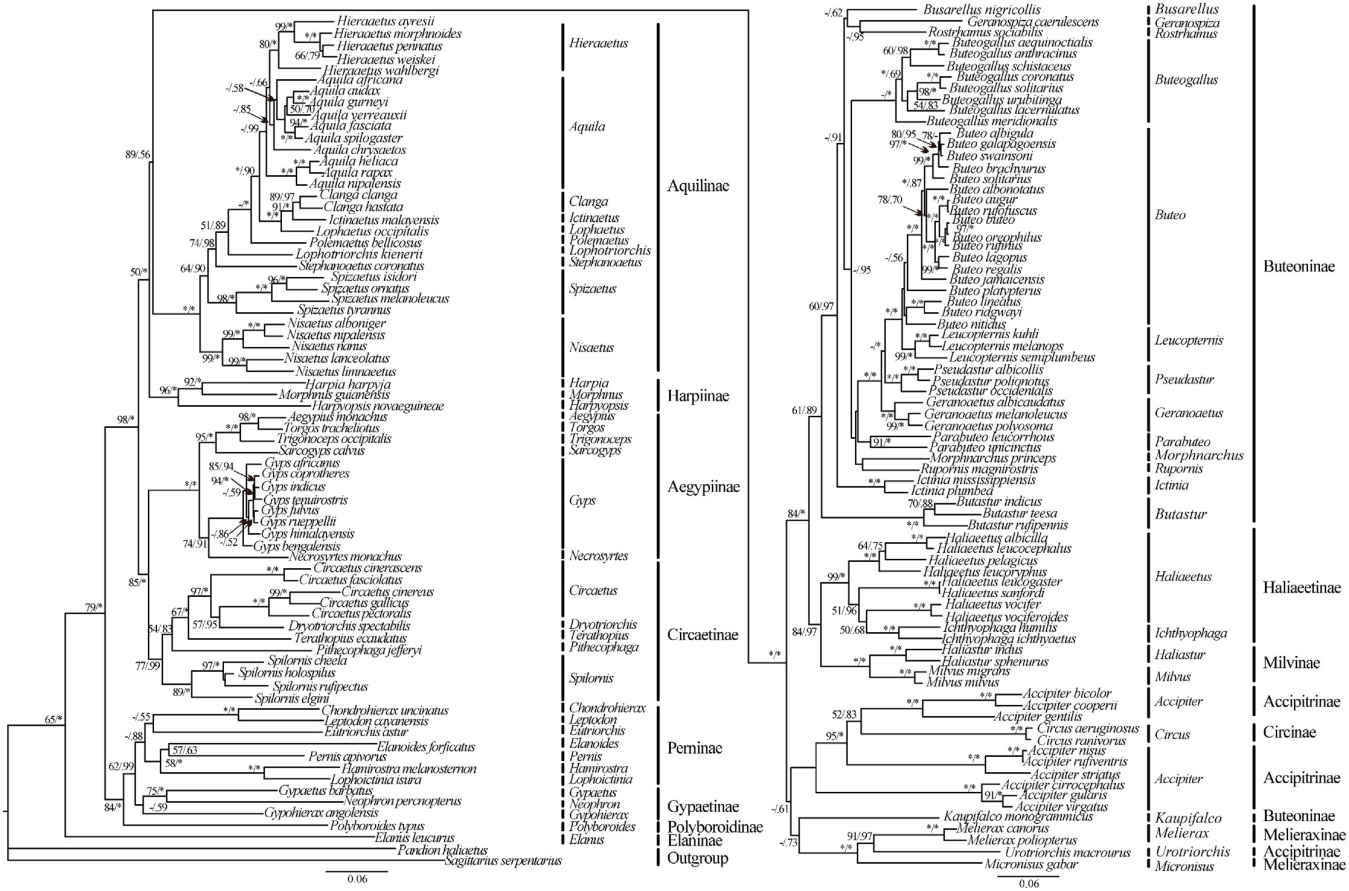
Phylogenetic analyses were performed using MT-ND2 and MT-CO1 genes for 60 genera (148 species) of Accipitridae, plus two species (*S*. *serpentarius and P*. *haliaetus*) as outgroups. ML bootstrap and Bayesian posterior probability values are presented above branches. Branch length and topology are taken from the ML analyses.

To test monophyly of Aquilinae, phylogenetic analysis was performed using MT-ND2 and MT-CYB genes for 60 genera and 148 species of Accipitridae, plus two species (*S*. *serpentarius* and *P*. *haliaetus*) as outgroups. There were a number of well-resolved basal clades within the family (Fig 5); the four most basal of these included: (1) a clade composed of monotypic genus *Elanus* (Elaninae), (2) a clade composed of Polyboroidinae, Perninae and Gypaetinae, (3) a clade that contained Circaetinae and Aegypiinae, and (4) a very large clade consisting of all remaining taxa. Within the last large clade, all species from aquiline eagles formed a robust monophyletic clade (Fig 5). Our results strongly support previous research on monophyly of Aquilinae[13,18]. Beyond recognizing the monophyly of Aquilinae, we also analysed phyloegentic relationships among and within subfamilies of Accipitridae. Our results were largely consistent with Jetz et al.’s phylogenetic trees[73] (the trees for this are available online at http://birdtree.org/), but differed in the positions of *Polyboroides typus*, *Accipiter striatus*, *Buteo nitidus*, and *Buteogallus lacernulatus* (Fig 5).

**Fig 6:**
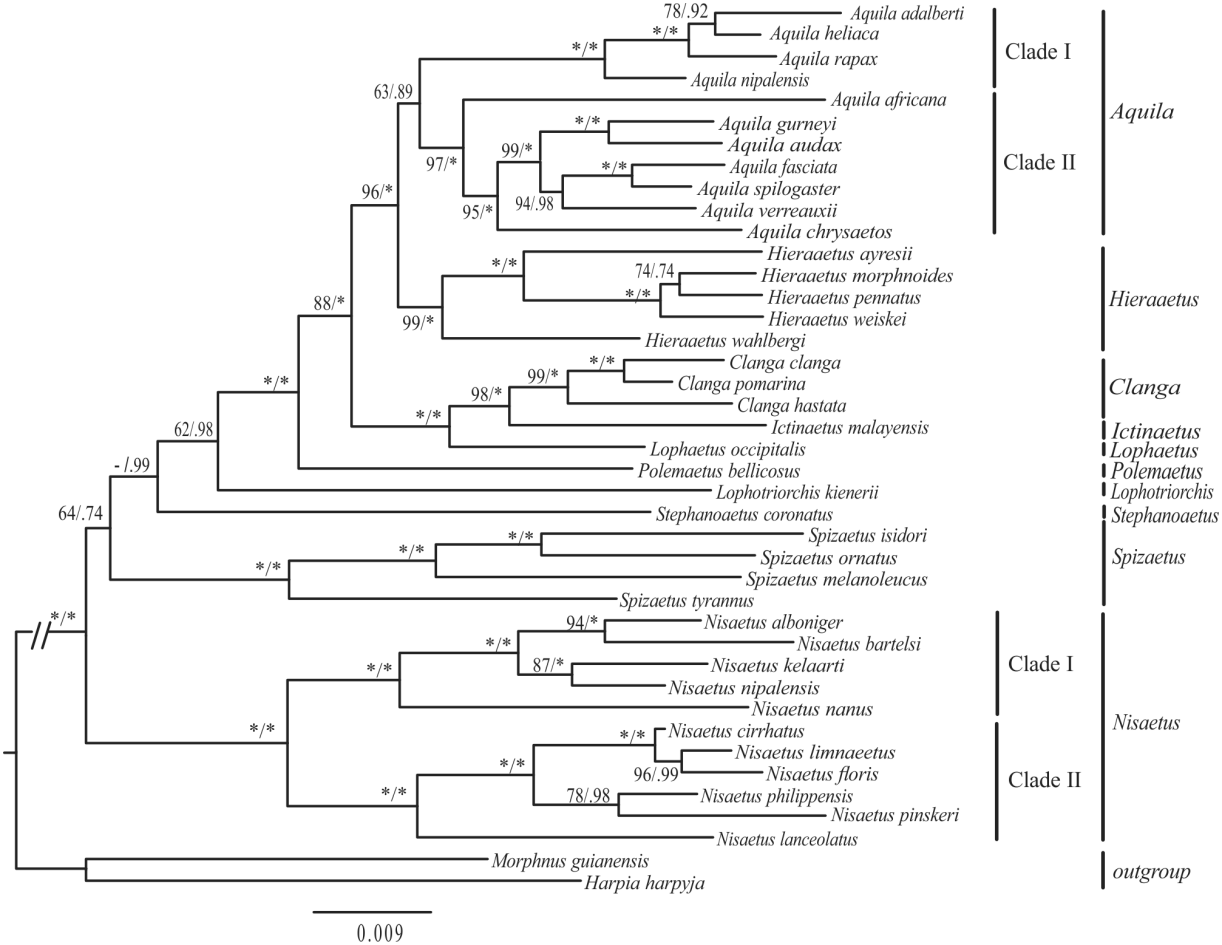
Inferred phylogenetic relationship among 39 species of Aquilinae plus two outgroups (*Morphnus guianensis* and *Harpia harpyja*). Phylogenetic tree of the relationships based on the nucleotide sequences of the 4 mitochondrial protein-coding genes(MT-CYB, MT-ND2, MT-CO1, CR) and 5 nuclear genes (RAG1, LDH, MYC, AK1, FIB7). Branch length and topology are taken from the Bayesian inference analyses.

To reassess monophyly of polytypic genera from Aquilinae, a phylogenetic analysis was carried out by combining mitochondrial and nuclear loci for 39 birds, representing all species and all genera from this subfamily. Some taxa are sampled for many loci, while other taxa are only represented by two loci (S3 Table). To a large-scale, sparse supermatrix of sequence data currently available, Burleigh et al. demonstrated that it be sufficient to produce a robust estimate of the avian tree of life [7]. So, in this analysis, we used the heterogeneous concatenated matrix data to infer phylogeny of Aquilinae. The ML and the BI methods generated identical topological trees (Fig 6). The monophyly of *Clanga*, *Hieraaetus*, *Nisaetus* and *Spizaetus* were strongly supported (>99% in ML, 1.00 in BI). The genus *Nisaetus*, previously the Old World birds of *Spizaetus*, was composed of two sister clades. Clade I included five species (*N*. *alboniger*, *N*. *bartelsi*, *N*. *kelaarti*, *N*. *nanus* and *N*. *nipalensis*), while Clade II harbored the remaining species of *Nisaetus*. The genus Aquila also consisted of two sister clades. Clade I was composed of four species (*A*. *adalberti*, *A*. *heliaca*, *A*. *rapax*, and *A*. *nipalensis*) (100% in ML, 1.00 in BI), while Clade II included the remaining birds of Aquila (97% in ML, 1.00 in BI). Notably, the monophyly of *Aquila* was weakly supported (63% in ML, 0.89 in BI). Based on above analyses, we recognize all currently polytypic genera of Aquilinae as monophyly.

## Conclusion

The complete mitochondrial sequences of *A*. *fasciata* and *B*. *lagopus* were found to be 18,513 and 18,559 bp with an A + T content of 54.2% and 55.0%, respectively. The genomes consist of 37 typical genes (13 energy pathway protein-coding genes, two ribosomal RNA genes and 22 transfer RNA genes)and two putative control regions (CR and ΨCR). The nucleotide variation across the entire genome sequence between the two accipitrid species was 19.07%. Obvious positive AT-skew and negative GC-skew biases were detected for all 12 PCGs encoded by the H strand, whereas the reverses were found in MT-ND6 encoded by the L strand. Six conserved sequence boxes in the Domain II, named boxes F, E, D, C, CSBa, and CSBb, respectively, were recognized in the CRs of *A*. *fasciata* and *B*. *lagopus*. The highest dN/dS was detected for the MT-ATP8 gene (0.29896) among Accipitridae, while the lowest for the MT-CO1 gene (0.01546). Mitophylogenetic analysis supported the robust monophyly of Accipitriformes, and Cathartidae was basal to the balance of the order. Moreover, our results indicate that the subfamily Aquilinae and all currently polytypic genera of this subfamily are monophyletic. These two novel mtDNA data will be useful in refining the phylogenetic relationships and evolutionary processes of Accipitriformes.

## Supporting Information

S1 Table Primers used in amplifying the mitogenomes of *Aquila fasciata* and *Buteo lagopus*. (DOC)

S2 Table GenBank accession numbers (MT-CYB, MT-ND2) for the 148 Accipitriformes species in this study. (XLS)

S3 Table GenBank accession numbers (MT-CYB, MT-ND2, MT-CO1, CR, RAG1, LDH, MYC, AK1 and FIB7) for 39 Aquilinae species in this study. (XLS)

S4 Table Genomic characteristics of Accipitriformes mtDNAs. (DOC)

S5 Table Localization and features of genes in the mitochondrial genomes of *Aquila fasciata* (AF) and *Buteo lagopus* (BL). (DOC)
