# Correction: The Mitochondrial Genomes of *Aquila fasciata* and *Buteo lagopus* (Aves, Accipitriformes): Sequence, Structure and Phylogenetic Analyses

**DOI:** 10.1371/journal.pone.0141037

**Published:** 2015-10-15

**Authors:** Lan Jiang, Juan Chen, Ping Wang, Qiongqiong Ren, Jian Yuan, Chaoju Qian, Xinghong Hua, Zhichun Guo, Lei Zhang, Jianke Yang, Ying Wang, Qin Zhang, Hengwu Ding, De Bi, Zongmeng Zhang, Qingqing Wang, Dongsheng Chen, Xianzhao Kan

There is an error in the seventh sentence of the Abstract. It should read: The highest dN/dS was detected for the MT-ATP8 gene (0.29896) among Accipitridae, while the lowest for the MT-CO1 gene (0.01546).

There is an error in the third sentence of the "Rates and patterns of mitochondrial gene evolution within Accipitridae" section of the Results and Discussion. It should read: In the protein coding region, the most variable region of the genomes by percent variable sites is MT-ATP8, followed by MT-ND4 and MT-ND6.

The images for Figs 3 and 4 are incorrectly switched. The image that appears as Fig 3 should be Fig 4, and the image that appears as Fig 4 should be Fig 3. The figure captions appear in the correct order. Please view the figures with their correct captions here.

**Fig 3:**
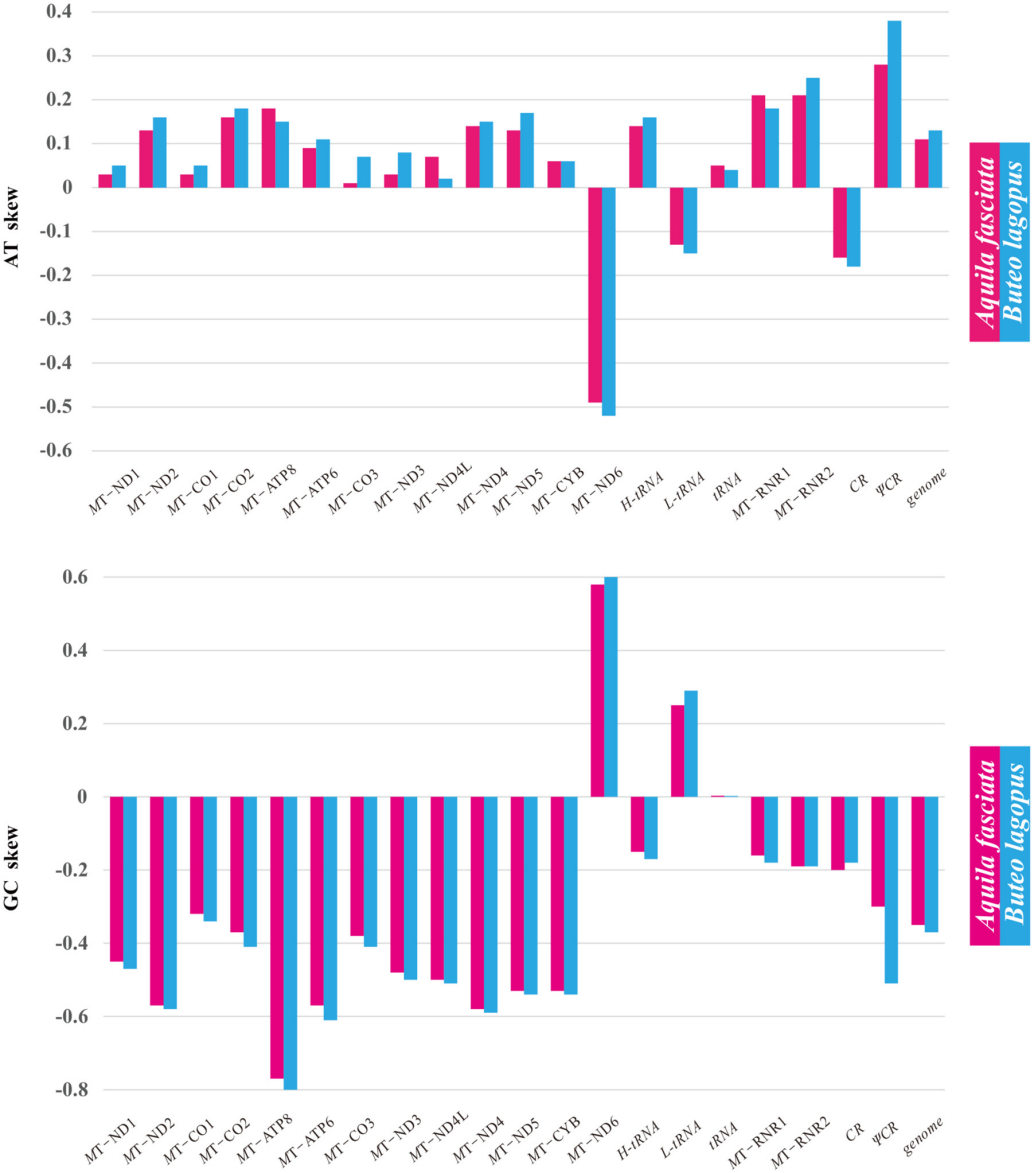
Base composition of *A*. *fasciata* and *B*. *lagopus* mitochondrial genomes. AT skew and GC skew are calculated for each protein-coding gene and other gene regions.

**Fig 4:**
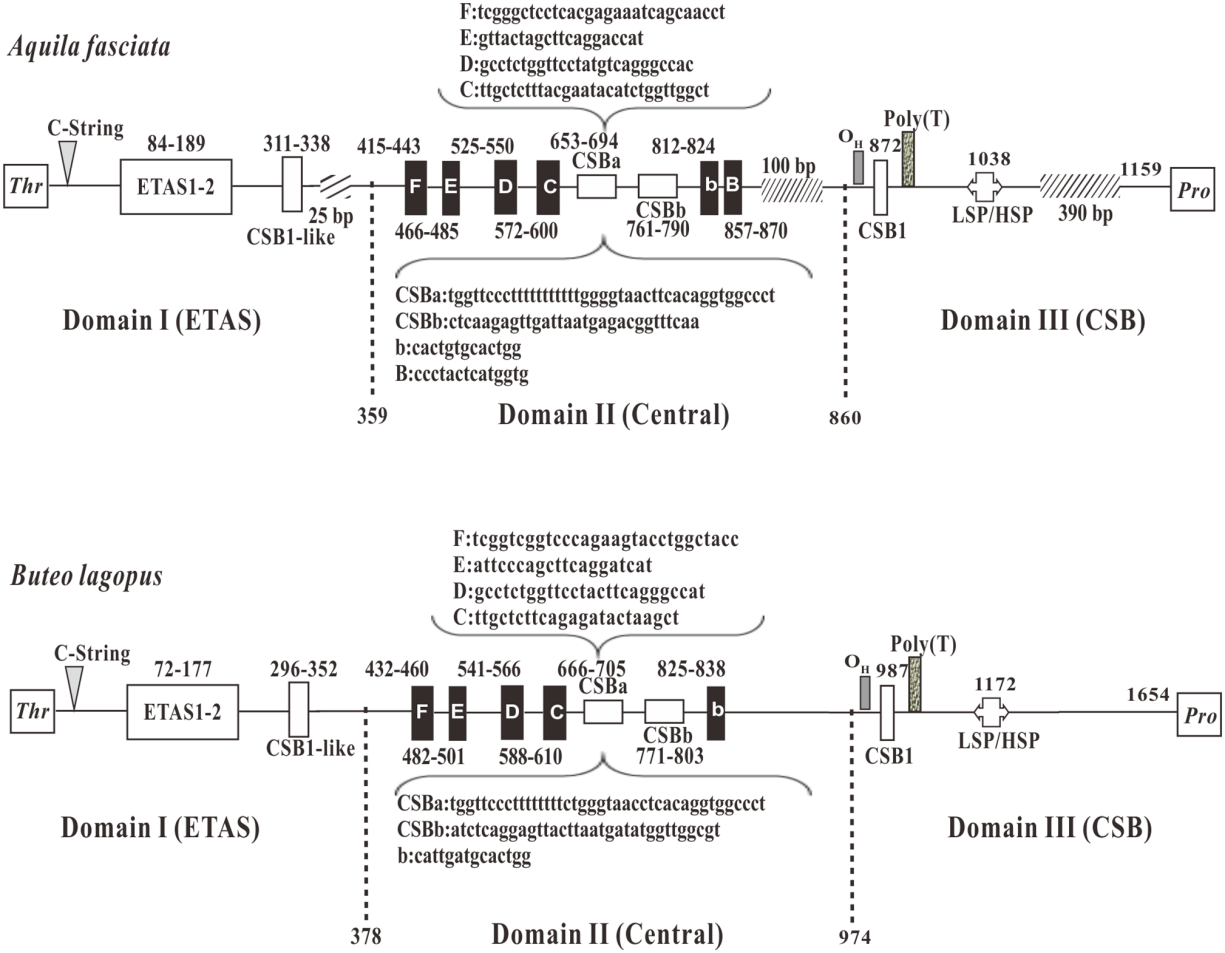
The structures of control region (CR) in mtDNA of *A*. *fasciata* and *B*.*lagopus*. Positions of the conserved boxes and the division into the three domains Domain I (ETAS), Domain II (Central), Domain III (CSB) are shown. ETAS = extended termination-associated sequences; F through B boxes = conserved sequence boxes in the central domain, CSBa is highly conserved stretches that vary in length, while CSBb is more variable; CSB = conserved sequence block; CSB-like = a sequence similar to the CSB; LSP = light-strand transcription promoter; HSP = heavy-strand transcription promoter; twill box means the comparison of two CRs, the lack of base number.
