# Supplementary material for: The Mitochondrial Genomes of Aquila fasciata and Buteo lagopus (Aves, Accipitriformes): Sequence, Structure and Phylogenetic Analyses
Source: PLoS One. 2015 Aug 21;10(8):e0136297. doi: 10.1371/journal.pone.0136297 (PMC4546579; doi:10.1371/journal.pone.0136297)
Supplement: S1 Table — (DOC) [file pone.0136297.s001.doc]

**S1 Table. Primers used in amplifying the mitogenomes of *Aquila fasciata* and *Buteo lagopus.***

| No. | Name | Sequences(5’-3’) | Size (bp) | Sources |
| --- | --- | --- | --- | --- |
| 1^a^ | KLACCMTabF1 | TCGGGCTGGTTCTCAATCTC | 20 | This study |
| 1^a^ | KLACCMTabR1 | GGTAGTTGGGTTGGTGTTGG | 20 | This study |
| 1^b^ | KLACCMTF1 | AGCCAACCAGTAGAACACCC | 20 | This study |
| 1^b^ | KLACCMTR1 | GGTGTCTTTGTGGCTCGGTT | 20 | This study |
| 2 ^a^ | KLACCMTabF2-1 | CCACCCAAATACACCATAAAC | 21 | This study |
| 2 ^a^ | KLACCMTabR2-1 | GGGCTTTGGTAGTATTGCTC | 20 | This study |
| 2 ^a^ | KLACCMTabF2-2 | CCCTACCAAACCATGTAACC | 20 | This study |
| 2 ^a^ | KLACCMTabF2-3 | CAATACTACCAAAGCCCTAA | 20 | This study |
| 2 ^a^ | KLACCMTabR2-2 | GGCTTTAGGCGTATCTTGGG | 20 | This study |
| 2^b^ | KLACCMTblF2-1 | ACCTCTATCACCAACTCCCA | 20 | This study |
| 2^b^ | KLACCMTblR2-1 | GGAATCAAGGGCATTTACAC | 20 | This study |
| 2^b^ | KLACCMTblR2-2 | AGTAAGGAATCAAGGGCA | 18 | This study |
| 2^b^ | KLACCMTblF2-2 | CCGTGAACTTATAACAAAGC | 19 | This study |
| 2^b^ | KLACCMTblR2-3 | TTTAGTTGGTCGTGGGCTGT | 20 | This study |
| 3^c^ | KLPASMTF3 | CCCACGGGTAATCAGCAGT | 19 |  |
| 3^c^ | KLPASMTR3 | ACTCTTTGTTGATGGCTGCT | 20 |  |
| 4 ^c^ | KLPASMTF4 | GAGGTGAAAAGCCAATCGAGC | 21 |  |
| 4 ^c^ | KLPASMTR4 | GCTAGGGAGAGGATTTGAACC | 21 |  |
| 5^c^ | KLPASMTF5 | AGTCCTACGTGATCTGAGTT | 20 |  |
| 5^c^ | KLPASMTR5 | GGCCCGATAGCTTGTTTAG | 19 |  |
| 6^c^ | KLPASMTF6 | GATAAAGTGAACATAGAGGT | 20 |  |
| 6^c^ | KLPASMTR6 | ATCGAAGCCCATCTGCCTA | 19 |  |
| 6^c^ | KLFALMTR6 | TCAAGGCCCATCTATCTAGGTT | 22 | This study |
| 7^c^ | KLPASMTF7 | GCCTTCAAAGCCTTAAACAA | 20 |  |
| 7^c^ | KLPASMTR7 | ATGGATAGGACGTAGTGGAA | 20 |  |
| 7^c^ | KLFALMTR7 | GTGTAGGCATCTGGGTAG | 18 | This study |
| 8^c^ | KLPASMTF8 | GAATGGACGTAGACACCCG | 19 |  |
| 8^c^ | KLPASMTR8 | CTGGTACATAGCTTCTTAAT | 20 |  |
| 8^c^ | KLFALMTF8 | GATAAAGTGAACATAGAGGT | 20 | This study |
| 8^c^ | KLFALMTR8 | AACCTAGATAGATGGGCCTTGA | 22 | This study |
| 9^c^ | KLFALMTF9 | ATGTTTTCTTGTTAGGTATAGG | 22 | This study |
| 9^c^ | KLFALMTR9 | ATCCTACCAGCAATTGTCCTC | 21 | This study |
| 10^c^ | KLFALMTF10 | ACGTCTTCGTCCTCCTACTAAG | 22 | This study |
| 10^c^ | KLFALMTR10 | GCTTTCTAGGCATAGTAGGGC | 21 | This study |
| 10^c^ | KLFALMTF10-2 | CAAGGAGGACTAGAGTGAGCAG | 22 | This study |
| 10^c^ | KLFALMTR10-2 | AGGTCAGTTTGGCGTAAGCAG | 21 | This study |
| 11^c^ | KLPASMTF11 | CCTCCTACAATGCTAAAAT | 19 |  |
| 11^c^ | KLPASMTR11 | CTTTCACTTGGATTTGCACC | 20 |  |
| 12^c^ | KLFALMTF12 | CTACTAACCGCCTCATACACC | 21 | This study |
| 12^c^ | KLPASMTF12 | AAAACCTTCTTACCTGCCGA | 20 |  |
| 12^c^ | KLPASMTR12 | CTTTTGAGTAGAATCCTGCT | 20 |  |
| 13^c^ | KLFALMTF13 | TCCTACTCATCCGCACCCA | 19 | This study |
| 13^c^ | KLFALMTR13 | TGTGTGGGCTACGGATGAG | 19 | This study |
| 14^c^ | KLPASMTF14 | CCCACACCATCAAACATCTC | 20 |  |
| 14^c^ | KLPASMTR14 | GGCTTACAAGACCAATG | 17 |  |

Note: ^a,b^ superscripts represent the primers for *A. fasciata* and *B. lagopus*, respectively and ^c^ indicates the conserved primers for both species.
