# Supplementary material for: The Mitochondrial Genomes of Aquila fasciata and Buteo lagopus (Aves, Accipitriformes): Sequence, Structure and Phylogenetic Analyses
Source: PLoS One. 2015 Aug 21;10(8):e0136297. doi: 10.1371/journal.pone.0136297 (PMC4546579; doi:10.1371/journal.pone.0136297)
Supplement: S4 Table — (DOC) [file pone.0136297.s004.doc]

**S4 Table**. Genomic characteristics of Accipitriformes mtDNAs

| Species | Heavy-strand | |  | Protein-coding genes | | | | |  | MT-RNR2 | |  | MT-RNR1 | |  | tRNA genes | |  | CR | |  | ΨCR | |
| --- | --- | --- | --- | --- | --- | --- | --- | --- | --- | --- | --- | --- | --- | --- | --- | --- | --- | --- | --- | --- | --- | --- | --- |
|  | Length  (bp) | AT% |  | Length  (bp) | AT%  (all) | AT%  (1st) | AT%  (2nd) | AT%  (3rd) |  | Length  (bp) | AT% |  | Length  (bp) | AT% |  | Length  (bp) | AT% |  | Length  (bp) | AT% |  | Length  (bp) | AT% |
| *Accipiter gentilis* | 18266 | 55.9 |  | 11397 | 54.2 | 49.7 | 58.3 | 55.1 |  | 1595 | 56.4 |  | 971 | 52.3 | | 1541 | 57.6 | | 1977 | 61.8 |  | 746 | 68.0 |
| *Accipiter nisus* | 18647 | 57.0 |  | 11400 | 55.0 | 49.8 | 58.1 | 57.8 |  | 1602 | 55.4 |  | 971 | 52.1 | | 1548 | 57.6 | | 2329 | 65.0 |  | 759 | 70.9 |
| *Accipiter soloensis* | 17900 | 55.7 |  | 11397 | 54.5 | 49.8 | 58.2 | 55.2 |  | 1597 | 56.1 |  | 969 | 51.4 | | 1549 | 57.4 | | 1276 | 58.5 |  | 1076 | 67.6 |
| *Accipiter virgatus* | 17952 | 54.9 |  | 11397 | 53.6 | 49.7 | 58.2 | 54.9 |  | 1600 | 55.4 |  | 972 | 51.7 | | 1548 | 57.1 | | 1308 | 58.1 |  | 1092 | 65.4 |
| *Aegypius monachus* | 17811 | 54.0 |  | 11400 | 52.5 | 48.6 | 57.2 | 52.1 |  | 1628 | 53.9 |  | 981 | 51.1 | | 1553 | 56.7 | | 1225 | 55.9 |  | 1025 | 68.0 |
| *Aquila chrysaetos* | 17332 | 53.3 |  | 11397 | 52.4 | 48.3 | 58.1 | 51.5 |  | 1537 | 54.3 |  | 968 | 49.2 | | 1551 | 57.1 | | 1158 | 56.2 |  | 627 | 61.9 |
| *Aquila fasciata* | 18513 | 54.2 |  | 11400 | 53.1 | 48.3 | 58.0 | 52.3 |  | 1594 | 53.5 |  | 971 | 50.2 | | 1550 | 56.7 | | 1159 | 57.2 |  | 1799 | 60.8 |
| *Buteo buteo* | 18674 | 55.2 |  | 11400 | 53.0 | 48.5 | 58.1 | 52.0 |  | 1598 | 55.0 |  | 972 | 51.7 | | 1547 | 57.0 | | 1672 | 63.3 |  | 1455 | 64.4 |
| *Buteo buteo burmanicus* | 18231 | 55.0 |  | 11397 | 53.0 | 48.5 | 58.0 | 53.0 |  | 1593 | 55.0 |  | 971 | 51.2 | | 1547 | 57.0 | | 1675 | 64.5 |  | 1018 | 64.2 |
| *Buteo lagopus* | 18559 | 55.0 |  | 11400 | 52.8 | 48.4 | 58.0 | 52.2 |  | 1594 | 55.0 |  | 973 | 51.6 | | 1546 | 57.0 | | 1654 | 63.7 |  | 1360 | 64.2 |
| *Nisaetus alboniger* | 17977 | 53.5 |  | 11397 | 51.7 | 48.1 | 57.0 | 58.8 |  | 1589 | 52.7 |  | 969 | 49.3 | | 1543 | 56.9 | | 1162 | 57.3 |  | 1273 | 66.8 |
| *Nisaetus nipalensis* | 17667 | 53.4 |  | 11397 | 51.9 | 48.3 | 58.1 | 49.4 |  | 1597 | 52.9 |  | 969 | 48.9 | | 1542 | 56.9 | | 1158 | 57.3 |  | 960 | 68.2 |
| *Spilornis cheela* | 18291 | 53.0 |  | 11400 | 51.4 | 48.1 | 58.3 | 48.5 |  | 1598 | 53.5 |  | 972 | 49.7 | | 1549 | 55.7 | | 1144 | 55.9 |  | 1532 | 62.0 |
| *Cathartes aura* | 16779 | 54.3 |  | 11406 | 53.5 | 49.3 | 58.3 | 53.3 |  | 1606 | 54.6 |  | 973 | 52.7 | | 1553 | 57.7 | | 1177 | 58.7 |  | — | — |
| *Pandion haliaetus* | 17864 | 55.0 |  | 11403 | 53.8 | 50.4 | 58.2 | 56.1 |  | 1581 | 54.6 |  | 971 | 51.9 | | 1542 | 57.1 | | 1156 | 56.1 |  | 1189 | 57.3 |
| *Sagittarius serpentarius* | 16773 | 54.6 |  | 11421 | 54.4 | 49.6 | 58.5 | 55.1 |  | 1610 | 53.6 |  | 972 | 51.8 | | 1560 | 56.9 | | 1117 | 58.0 |  | — | — |
