# Supplementary material for: The Mitochondrial Genomes of Aquila fasciata and Buteo lagopus (Aves, Accipitriformes): Sequence, Structure and Phylogenetic Analyses
Source: PLoS One. 2015 Aug 21;10(8):e0136297. doi: 10.1371/journal.pone.0136297 (PMC4546579; doi:10.1371/journal.pone.0136297)
Supplement: S5 Table — (DOC) [file pone.0136297.s005.doc]

**S5 Table.** Localization and features of genes in the mitochondrial genomes of *Aquila fasciata* (AF) and *Buteo lagopus* (BL)

| Gene/region | Strand | Position | |  | Start/Stop Codon | |  | Anticodon |  | Intergenic nucleotides nununucleotides | |
| --- | --- | --- | --- | --- | --- | --- | --- | --- | --- | --- | --- |
|  |  | AF | BL |  | AF | BL |  |  |  | AF | BL |
| MT-TF | H | 1-70 | 1-70 |  |  |  |  | GAA |  | 0 | 0 |
| MT-RNR1 | H | 71-1041 | 71-1043 |  |  |  |  |  |  | 0 | 0 |
| MT-TV | H | 1042-1113 | 1044-1114 |  |  |  |  | TAC |  | 0 | 0 |
| MT-RNR2 | H | 1114-2707 | 1115-2708 |  |  |  |  |  |  | 0 | 0 |
| MT-TL1 | H | 2708-2781 | 2709-2782 |  |  |  |  | TAA |  | 9 | 9 |
| MT-ND1 | H | 2791-3768 | 2792-3769 |  | ATG/AGG | ATG/AGG |  |  |  | -2 | -2 |
| MT-TI | H | 3767-3838 | 3768-3839 |  |  |  |  | GAT |  | 15 | 15 |
| MT-TQ | L | 3852-3922 | 3853-3923 |  |  |  |  | TTG |  | -1 | -1 |
| MT-TM | H | 3922-3990 | 3923-3991 |  |  |  |  | CAT |  | 0 | 0 |
| MT-ND2 | H | 3991-5031 | 3992-5032 |  | ATG/TAG | ATG/TAG |  |  |  | 0 | 0 |
| MT-TW | H | 5030-5101 | 5031-5102 |  |  |  |  | TCA |  | 1 | 1 |
| MT-TA | L | 5103-5171 | 5104-5172 |  |  |  |  | TGC |  | 2 | 2 |
| MT-TN | L | 5174-5246 | 5175-5247 |  |  |  |  | GTT |  | 2 | 2 |
| MT-TC | L | 5249-5315 | 5250-5316 |  |  |  |  | GCA |  | -1 | -1 |
| MT-TY | L | 5315-5385 | 5316-5386 |  |  |  |  | GTA |  | 1 | 1 |
| MT-CO1 | H | 5387-6937 | 5388-6938 |  | GTG/AGG | GTG/AGG |  |  |  | -9 | -9 |
| MT-TS1 | L | 6929-7002 | 6930-7003 |  |  |  |  | TGA |  | 5 | 4 |
| MT-TD | H | 7008-7076 | 7008-7076 |  |  |  |  | GTC |  | 2 | 2 |
| MT-CO2 | H | 7079-7762 | 7079-7762 |  | ATG/TAA | ATG/TAA |  |  |  | 1 | 1 |
| MT-TK | H | 7764-7834 | 7764-7831 |  |  |  |  | TTT |  | 1 | 1 |
| MT-ATP8 | H | 7836-8003 | 7833-8000 |  | ATG/TAA | ATG/TAA |  |  |  | -10 | -10 |
| MT-ATP6 | H | 7994-8677 | 7991-8674 |  | ATG/TAA | ATG/TAA |  |  |  | -1 | -1 |
| MT-CO3 | H | 8677-9460 | 8674-9457 |  | ATG/T- | ATG/T- |  |  |  | 0 | 0 |
| MT-TG | H | 9461-9529 | 9458-9526 |  |  |  |  | TCC |  | 0 | 0 |
| MT-ND3 | H | 9530-9881 | 9527-9877 |  | ATC/TAA | ATC/TAA |  |  |  | 7 | 4 |
| MT-TR | H | 9889-9957 | 9882-9950 |  |  |  |  | TCG |  | 1 | 1 |
| MT-ND4L | H | 9959-10255 | 9952-10248 |  | ATG/TAA | ATG/TAA |  |  |  | -7 | -7 |
| MT-ND4 | H | 10249-11626 | 10242-11619 |  | ATG/T- | ATG/T- |  |  |  | 0 | 0 |
| MT-TH | H | 11627-11696 | 11620-11689 |  |  |  |  | GTG |  | 0 | 0 |
| MT-TS2 | H | 11697-11762 | 11690-11755 |  |  |  |  | GCT |  | 0 | 0 |
| MT-TL2 | H | 11763-11833 | 11756-11826 |  |  |  |  | TAG |  | 0 | 0 |
| MT-ND5 | H | 11834-13651 | 11827-13644 |  | ATG/TAA | GTG/AGA |  |  |  | 11 | 17 |
| MT-CYB | H | 13663-14805 | 13662-14804 |  | ATG/TAA | ATG/TAA |  |  |  | 4 | 2 |
| MT-TT | H | 14810-14879 | 14807-14876 |  |  |  |  | TGT |  | 0 | 0 |
| CR | H | 14880-16038 | 14877-16530 |  |  |  |  |  |  | 0 | 0 |
| MT-TP | L | 16039-16108 | 16531-16600 |  |  |  |  | TGG |  | 13 | 6 |
| MT-ND6 | L | 16122-16640 | 16607-17125 |  | ATG/TAG | ATG/TAG |  |  |  | 3 | 3 |
| MT-TE | L | 16644-16714 | 17129-17199 |  |  |  |  | TTC |  | 0 | 0 |
| ΨCR | H | 16715-18513 | 17200-18559 |  |  |  |  |  |  | 0 | 0 |

Note: ^a^ “-” Indicates termination codons completed via polyadenylation. ^b^Negative values represent overlapping nucleotides
